# Correction

**DOI:** 10.1080/19491034.2024.2436700

**Published:** 2024-12-05

Article title: VPS4B orchestrates response to nuclear envelope stress by regulating ESCRT-III dynamics in glioblastoma

Authors: Z. Wu., I. Omura., A. Saito., K. Imaizumi., and Y. Kamikawa

Journal: Nucleus

DOI: https://doi.org/10.1080/19491034.2024.

This article was published earlier with an error in the caption of figures. This has now been corrected and republished in the original article.

**Figure 1. Differential response to NE stress between GBM cell lines.**

**A**. Schematic representation of the induction of NE stress under constricted migration using Transwell.

**B**. Immunofluorescence staining analysis of lamin B1 (magenta) and γH2A.X (green) of U251MG and U87MG cells on the bottom side of a Transwell. Overlay indicates merged images with DNA (blue) staining. Arrowheads represent the accumulation of γH2A.X. Scale bars: 5 μm.

**C**. Quantification of the proportion of the cells with γH2A.X accumulation indicated in (B). Bars and error bars represent the mean values and standard deviations from three independent experiments. Statistical significance of the difference was determined using Fisher’s exact test. ***: *p *< 0.005. Total cell numbers for each cell line from three independent experiments: n =241, U251MG, and n =163, U87MG.

**D**, Localization of mSCI3-Progerin (green) in U251-Tet-mSCI3-Progerin (upper) and U87-Tet-mSCI3-Progerin (lower) 48 hours after cultivation in the absence of (DOX-) and the presence of DOX (DOX+). Overlay indicates merged images with DNA (blue) staining. Scale bars: 5 μm.

**E**. Quantification of nuclear circularity of U251- and U87-Tet-mSCI3-Progerin cells in the absence of or presence of DOX indicated in (D). Bars and error bars represent the mean values and standard deviations from three independent experiments. The statistical significance of the difference was determined using Brunner-Munzel test. ***: *p*<0.005. n.s.: *p*>0.05. Total cell numbers for each cell line from three independent experiments: n =138, DOX- U251; n = 241, DOX+ U251; n=166, DOX- U87MG; n = 158 DOX+ U87MG.

**Figure 2. Reduced expression of VPS4B in U251MG.**

**A, B**. Western blotting analysis of NE repair factors and the quantification of band intensity of VPS4B in U251MG and U87MG. β-Actin was used as the loading control. Bars and error bars represent the mean values and standard deviations from three independent experiments. Statistical significance of the difference was determined using Student’s *t* test. *: *p*<0.05.

**C**. Real-time qPCR analysis of mRNA level of VPS4B in U251MG and U87MG. β-Actin was used as the internal control. Bars and error bars represent the mean values and standard deviations from three independent experiments. Statistical significance of the difference was determined using Student’s *t* test. **: *p*<0.01.

**Figure 3. VPS4B suppresses DNA damage induced by NE stress.**

**A**. Western blotting analysis of VPS4B in U87MG transfected with non-targeting siRNA (siCon) or VPS4B targeting siRNA (siVPS4B). β-Actin was used as the loading control.

**B**. Immunofluorescence staining analysis of lamin B1 (magenta) and γH2A.X (green) of U87MG cells transfected with non-targeting siRNA (siCon) or VPS4B targeting siRNA (siVPS4B) at the bottom side of a Transwell. Overlay indicates merged images with DNA (blue) staining. Arrowhead indicates the prominent signal of γH2A.X accumulation. Scale bars: 5 μm.

**C**. Quantification of the proportion of the cells with γH2A.X accumulation shown in (B). Bars and error bars represent the mean values and standard deviations from three independent experiments. Statistical significance of the difference was determined using Fisher’s exact test. **: *P*<0.01. Total cell numbers for each condition from three independent experiments: n = 393, siCon; and n = 396, siVPS4B.

**D**. Western blotting analysis of Tet-VPS4B-mVenus in the absence (DOX−) or presence of DOX (DOX+). β-Actin was used as the loading control.

**E**. Immunofluorescence of γH2A.X (magenta) together with the signal of mVenus (green) derived from VPS4B-mVenus at the bottom side of Transwell in the absence of (DOX−) or presence of DOX (DOX+). Overlay indicates merged images with DNA (blue) staining. Arrowhead indicates the prominent signal of γH2A.X. Scale bars: 5 μm.

**F**. Quantification of the proportion of the cells with γH2A.X accumulation in Tet-VPS4B-mVenus cells indicated in (E). Bars and error bars represent the mean values and standard deviations from three independent experiments. Statistical significance of the difference was determined using Fisher’s exact test. ***: *p*<0.005. Total cell numbers for each condition from three independent experiments: n = 109, DOX−; and n = 114, DOX+.

**Figure 4. Alteration of VPS4B localization upon mechanical NE stress.**

**A**. Schematic representation of the cellular compression assay to induce NE rupture.

**B**. Localization of VPS4B-mVenus (green) in U251-Tet-VPS4B-mVenus cells in the presence of DOX under normal conditions (Normal) and just after cellular compression (Pressed). Overlay indicates merged images with DNA (blue) staining. Scale bars: 5 μm.

**C**. Quantification of the Tet-VPS4B-mVenus cells with the ratio of U251-VPS4B-mVenus signal intensity of the nucleus to that of the cytoplasm is larger than 1.5 under normal conditions (Normal) or just after cellular compression (Pressed) shown in (B). Bars and error bars represent the mean values and standard deviations from three independent experiments. Statistical significance of the difference was determined using Fisher’s exact test. **: *p *< 0.01. Total cell numbers for each condition from three independent experiments: n = 26, Normal; and n = 18, Pressed.

**D**. Localization of VPS4B-mVenus (green) and immunofluorescence of Lap2β (magenta) in Tet-VPS4B-mVenus in the presence of DOX at 0 min (0 min) and 30 min (30 min) after cellular compression. Overlay indicates merged images with DNA (blue) staining. Arrowheads represent the damaged NE where VPS4B is accumulated. Scale bars: 5 μm.

**E**. Quantification of the accumulation of VPS4B-mVenus in U251-Tet-VPS4B-mVenus cells at 0 min (0 min) and 30 min (30 min) after cellular compression indicated in (D). Bars and error bars represent the mean values and standard deviations from three independent experiments. The statistical significance of the difference was determined using Fisher’s exact test. ***: *P*<0.005. Total cell numbers for each condition from three independent experiments: n =112, 0 min; and n =85, 30 min.

**Figure 5. Dynamics of NE repair factors during the response to NE stress.**

**A**. Localization of VPS4B-mVenus (green) and immunofluorescence of BAF (magenta) in U251-Tet-VPS4B-mVenus cells in the presence of DOX at 0 min (0 min) and 30 min (30 min) after cellular compression. Overlay indicates merged images with DNA (blue) staining. Arrowheads represent the accumulation of BAF or VPS4B. Scale bars: 5 μm.

**B**. Quantification of the accumulation of BAF in U251-VPS4B-mVenus positive cells indicated in (A). Bars and error bars represent the mean values and standard deviations from three independent experiments. The statistical significance of the difference was determined using Fisher’s exact test. ***: *P*<0.005. Total cell numbers for each condition from three independent experiments: n =101, 0 min; and n =101, 30 min.

**C**. Immunofluorescence of CHMP7 (magenta) in U251MG cells under normal conditions (Normal) and just after cellular compression (Pressed). Overlay indicates merged images with DNA (blue) staining. Scale bars: 5 μm.

**D**. Quantification of the nuclear accumulation of CHMP7 (signal intensity of the nucleus to that of the cytoplasm > 1.5) in U251MG cells under normal conditions (Normal) and just after cellular compression (Pressed) in (C). Bars and error bars represent the mean values and standard deviations from three independent experiments. Statistical significance of the difference was determined using Fisher’s exact test. ***: *p *< 0.005. Total cell numbers for each condition from three independent experiments: n = 26, Normal; and n = 29, Pressed.

**E**. Localization of VPS4B-mVenus (green) and immunofluorescence of CHMP7 (magenta) in U251-Tet-VPS4B-mVenus cells in the presence of DOX at 0 min (0min) or 30 min (30min) after cellular compression. Overlay indicates merged images with DNA (blue) staining. Arrowheads represent the NE where VPS4B and CHMP7 are accumulated. Scale bars: 10 μm.

**F**. Quantification of the colocalization of CHMP7 and VPS4B in damaged U251-Tet-VPS4B-mVenus cells in the presence of DOX at 0 min (0 min) and 30 min (30 min) after cellular compression shown in (E). Bars and error bars represent the mean values and standard deviations from three independent experiments. The statistical significance of the difference was determined using Fisher’s exact test. ***: *P*<0.005. Total cell numbers for each condition from three independent experiments: n =71, 0 min; and n =30, 30 min.

**Figure 6. VPS4B regulates the dynamics of CHMP7 during response to NE stress and enhances NE repair.**

**A**. Localization of VPS4B-mVenus (green) and immunofluorescence of CHMP7 (magenta) at 0 min, 30 min, and 60 min after cellular compression. U251-Tet-VPS4B-mVenus cells were subjected to the compression in the absence of (DOX-) or presence of DOX (DOX +). Overlay indicates merged images with DNA (blue) staining. Scale bars: 5 μm.

**B**. Quantification of the accumulation of CHMP7 in U251-Tet-VPS4B-mVenus cells in the absence of (DOX−) or presence of DOX (DOX+) in different time points (0, 30, and 60 min after cellular compression) shown in (A). Bars and error bars represent the mean values and standard deviations from three independent experiments. Statistical significance of the difference was determined using Fisher’s exact test. n.s.: *p* > 0.05, *p<0.05. Total cell numbers for each condition from four independent experiments: n = 100, DOX−, 0 min; and n =116, DOX+, 0 min. n = 91, DOX−, 30 min; and n =94, DOX+, 30 min. n = 125, DOX−, 60 min; and n =108, DOX+, 60 min.

**C**. Dynamics of the repair of NE rupture induced by laser irradiation in control (siCon) and VPS4B-depleted (siVPS4B) U87MG cells. Snapshot images of time-lapse imaging of mSCI-BAF and 2XtGFP-NLS were represented. Interval and duration were 5 and 55 min, respectively. Arrowheads indicate the accumulation of mSCI-BAF. Scale bars: 5μm.

**D**. Quantification of the persistent accumulation of mSCI-BAF shown in (C). The percentiles of the cells positive for mSCI-BAF accumulation at the irradiated site 55min after treatment were represented. *p*-value was calculated using Fisher’s exact test. Total cell numbers for each condition from two or three independent experiments: n=11; non-targeting siRNA (siCon) and n=11; siRNA targeting for VPS4B (siVPS4B).

**E**. Quantification of the ratio of 2XtGFP-NLS signal intensity of the nucleus to that of cytoplasm shown in (C). The values before laser irradiation were set at 1.0. The dots and error bars represent mean values and standard deviation from two or three independent experiments. Total cell numbers for each cell line: n =11 , siCon; and n = 10, siVPS4B. Statistical significance of the difference 55min after irradiation was determined by Brunner-Munzel test. *: *p*<0.05

**Figure 7. Recovery of the nuclear lamina after mechanical compression and the interaction between VPS4B and CHMP7.**

**A**. Localization of VPS4B-mVenus (green) and immunofluorescence of lamin B1 (magenta) at 0 min, 30 min, and 60 min after cellular compression. U251-Tet-VPS4B-mVenus cells were subjected to the compression in the absence of (DOX−) or presence of DOX (DOX +). Overlay indicates merged images with DNA (blue) staining. Scale bars: 5 μm.

**B**. Quantification of the gap of the nuclear lamina shown in (A). Bars and error bars represent the mean values and standard deviations from three independent experiments. Statistical significance of the difference was determined using Brunner-Munzel test. n.s.: *p* > 0.05, *** *p*<0.005. Total cell numbers for each condition from three independent experiments: n = 44, 0 min (DOX-); n = 51, 0 min (DOX+); n =48, 30 min (DOX-); n = 59, 30 min (DOX+); n = 54, 60 min (DOX-); n = 57, 60 min (DOX+); n = 49, 120 min (DOX-); n = 53, 120 min (DOX+).

**C**. Interaction between endogenous CHMP7 and an ATPase deficient mutant of VPS4B^E235Q^. Co-immunoprecipitation was performed using the lysate fromTet-VPS4B^E235Q^-mVenus cells both in the absence (DOX−) of and presence of the DOX (DOX+) with anti-CHMP7 antibodies. Normal Rabbit IgG was used as the negative control. VPS4B^E235Q^-mVenus was detected using anti-GFP antibodies. Co-immunoprecipitation of VPS4B^E235Q^-mVenus with endogenous CHMP7 was also observed in additional two independent experiments.

